# *N*-Methyl-*N*-styrylcinnamamide (lansamide) from *Clausena lansium* in Vietnam

**DOI:** 10.1107/S1600536809009611

**Published:** 2009-03-19

**Authors:** Peter Luger, Manuela Weber, Tran Dinh Thang, Hoang Van Luu, Nguyen Xuan Dung

**Affiliations:** a Institut für Chemie und Biochemie-Kristallographie, Fachbereich Biologie-Chemie-Pharmazie der Freien Universität, Fabeckstrasse 36a, 14195 Berlin, Germany; b Faculty of Chemistry, Vinh University, 182-Le Duan, Vinh City, Nghean Province, Vietnam; c Faculty of Chemistry, College of Natural Sciences, Hanoi National University, 19-Le Thanh Tong Street, 10 000 Hanoi, Vietnam

## Abstract

The title compound, C_18_H_17_NO, was isolated from the seeds of *Clausena lansium* (wampee) (Rutaceae). The X-ray crystal structure analysis confirmed its chemical identity and revealed that it is solvent-free, in contrast to the previously reported monohydrate [Huang, Ou & Tang (2006 ▶). *Acta Cryst*. E**62**, o1987–o1988]. The mol­ecular structures are practically identical but the mol­ecules pack differently. In contrast to the monohydrate in which the water molecule generates two hydrogen bonds, no such intermolecular contacts are present in the title compound. The dihedral angle between the cinnamamide and the styryl group is 53.1 (1)°.

## Related literature

For the structure of the monohydrate, see: Huang *et al.* (2006 ▶). For medicinal applications, see: Loi (2001 ▶).
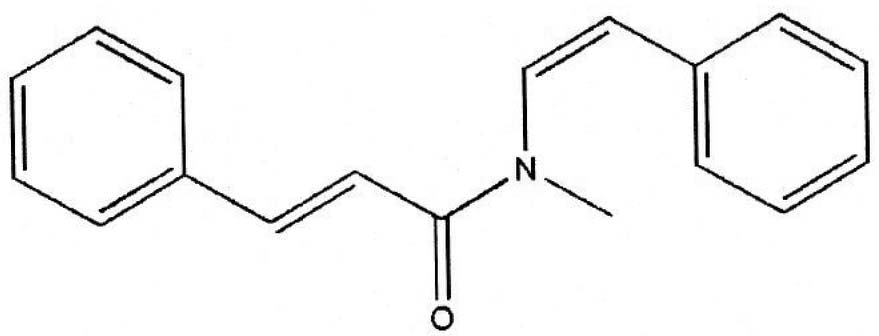


## Experimental

### 

#### Crystal data

C_18_H_17_NO
*M* *_r_* = 263.33
Triclinic, 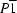

*a* = 6.356 (1) Å
*b* = 9.265 (2) Å
*c* = 13.073 (3) Å
α = 80.45 (3)°
β = 77.22 (3)°
γ = 78.13 (3)°
*V* = 728.9 (3) Å^3^
*Z* = 2
Mo *K*α radiation
μ = 0.07 mm^−1^
*T* = 298 K
0.60 × 0.60 × 0.55 mm

#### Data collection

Bruker SMART CCD area-detector diffractometer
Absorption correction: none
25654 measured reflections
5327 independent reflections
3962 reflections with *I* > 2σ(*I*)
*R*_int_ = 0.023

#### Refinement

*R*[*F* ^2^ > 2σ(*F* ^2^)] = 0.060
*wR*(*F* ^2^) = 0.190
*S* = 1.02
5327 reflections
182 parameters
H-atom parameters constrained
Δρ_max_ = 0.36 e Å^−3^
Δρ_min_ = −0.14 e Å^−3^

### 

Data collection: *SMART* (Bruker, 2004 ▶); cell refinement: *SAINT* (Bruker, 2004 ▶); data reduction: *SAINT*; program(s) used to solve structure: *SHELXS97* (Sheldrick, 2008 ▶); program(s) used to refine structure: *SHELXL97* (Sheldrick, 2008 ▶); molecular graphics: *ORTEPIII* (Burnett & Johnson, 1996 ▶) and *SCHAKAL99* (Keller & Pierrard, 1999 ▶); software used to prepare material for publication: *SHELXL97*.

## Supplementary Material

Crystal structure: contains datablocks I, global. DOI: 10.1107/S1600536809009611/tk2394sup1.cif

Structure factors: contains datablocks I. DOI: 10.1107/S1600536809009611/tk2394Isup2.hkl

Additional supplementary materials:  crystallographic information; 3D view; checkCIF report

## Figures and Tables

**Fig. 1.**
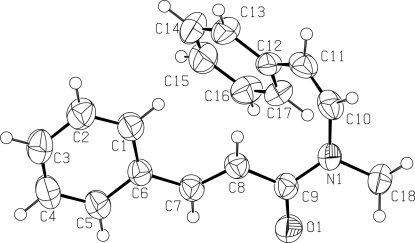
Molecular structure of (I) with atom numbering scheme, displacement ellipsoids are shown at the 50% probability level.

**Fig. 2.**
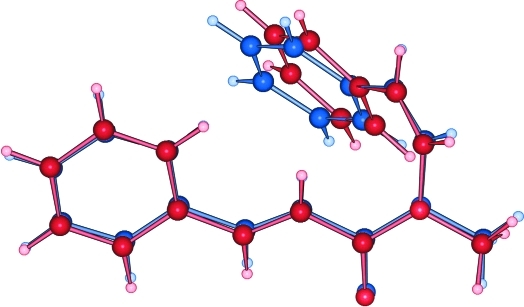
Superposition of (I) (red) and the monohydrate (blue) forms of lansamide.

**Fig. 3.**
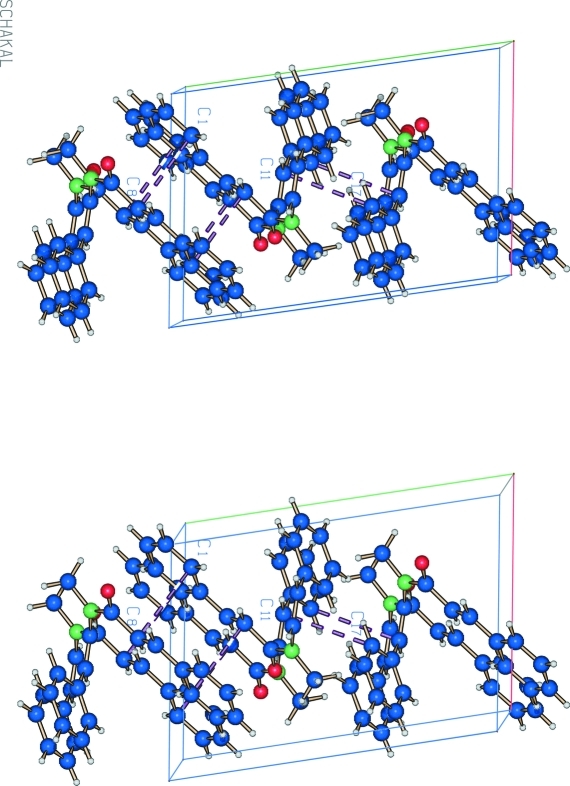
Stereo drawing of packing in (I) shown in projection onto the yz-plane. The C1···C8 and C11···C17 contacts are shown by dashed lines.

## supplementary crystallographic information

### Comment

The title compound *N*-methyl-*N*-styrylcinnamamide [*N*-methyl-3-phenyl-*N*-(2-phenylethenyl)-2-propenamide] (I) was isolated from the seeds of Clausena lansium (wampee) (Rutaceae). The leaves have been used as a folk medicine for the treatment of coughs, asthma and gastrointestinal diseases. The fruit is used for digestive disorders and the seeds are used for gastro-intestinal diseases such as acute and chronic gastrointestinal, ulcers, *etc*. (Loi, 2001).

The X-ray structure of the monohydrate of (I) was reported recently (Huang *et al.*,  2006) wherein it was reported that "the corresponding anhydrous compound is a pale-yellow liquid at room temperature". Surprisingly, we could grow pale-yellow crystals of the water-free form from n-hexane and its X-ray structure, which is subject of this study, was carried out to confirm its chemical identity.

The molecular structure of (I) is shown in Fig. 1 and its superposition with the molecule found in the monohydrate structure is shown in Fig. 2. Both structures are practically identical with a small difference of 10° in the rotation of the styryl phenyl ring along the bond C11—C12: torsion angle C10—C11—C12—C13= 148.7 (1)° for (I) and 139.0 (3)° for the monohydrate. Bond lengths and angles, which are all in the expected ranges, have an average difference of 0.007 Å and 0.4° between (I) and the monohydrate.

The triclinic lattice of (I) is illustrated in Fig. 3. In contrast to the monohydrate where the water molecule generates two hydrogen bonds in a body centered tetragonal lattice, no such intermolecular contacts are present in (I). Nevertheless, the packing in the triclinic lattice shows some characteristic features. The molecule consists of two planar fragments, the cinnamamide and the styryl group, forming an interplanar angle of 53.1 (1)°. In molecular pairs related by the inversion centre at (1/2, 1/2, 1), the cinnamamide fragments are aligned in parallel planes with a shortest contact distance of C atoms of adjacent planes being C1···C8 = 3.664 (3) Å. Such an arrangement of cinnamamide groups was also observed in the monohydrate structure. The styryl groups also form co-planar planes for molecules related by the inversion centre at (1/2, 1/2, 1/2), the shortest distance between C atoms of adjacent planes is C11···C17 = 3.730 (3) Å (see dashed lines in Fig. 3). This arrangement of styryl groups was not observed in the monohydrate structure.

### Experimental

The dried seeds of C. lansium (3,0 kg) were powdered and extracted with MeOH at room temperature, and the combined extracts were concentrated under reduced pressure to give a deep-brown syrup (160 g). This was partitioned between H_2_O and n-hexane. The n-hexane-soluble residue (85 g) was chromatographed over a silica gel column, which developed by gradient elution with n-hexane and increasing concentrations of Me_2_CO to afford forty fractions. Fractions were combined on their TLC. After standing for several day, fractions 9–11 recrystallized from n-hexane to afford pale-yellow lansamide (2554 mg).

### Refinement

Carbon-bound H-atoms were placed in calculated positions (C—H 0.93 to 0.96 Å) and were included in the refinement in the riding model approximation, with *U*(H) set to 1.2 to 1.5*U*(C).

### Crystal data

**Table:**

C_18_H_17_NO	*Z* = 2
*M**_r_* = 263.33	*F*(000) = 280
Triclinic, *P*1	*D*_x_ = 1.200 Mg m^−^^3^
Hall symbol: -P 1	Mo *K*α radiation, λ = 0.71073 Å
*a* = 6.356 (1) Å	Cell parameters from 5327 reflections
*b* = 9.265 (2) Å	θ = 2.3–33.3°
*c* = 13.073 (3) Å	µ = 0.07 mm^−^^1^
α = 80.45 (3)°	*T* = 298 K
β = 77.22 (3)°	Block, yellow
γ = 78.13 (3)°	0.60 × 0.60 × 0.55 mm
*V* = 728.9 (3) Å^3^	

### Data collection

**Table:**

Bruker SMART CCD area-detector diffractometer	3962 reflections with *I* > 2σ(*I*)
Radiation source: fine-focus sealed tube	*R*_int_ = 0.023
graphite	θ_max_ = 33.3°, θ_min_ = 2.3°
ω scans	*h* = −9→9
25654 measured reflections	*k* = −13→14
5327 independent reflections	*l* = 0→20

### Refinement

**Table:**

Refinement on *F*^2^	Primary atom site location: structure-invariant direct methods
Least-squares matrix: full	Secondary atom site location: difference Fourier map
*R*[*F*^2^ > 2σ(*F*^2^)] = 0.060	Hydrogen site location: inferred from neighbouring sites
*wR*(*F*^2^) = 0.190	H-atom parameters constrained
*S* = 1.02	*w* = 1/[σ^2^(*F*_o_^2^) + (0.0892*P*)^2^ + 0.1313*P*] where *P* = (*F*_o_^2^ + 2*F*_c_^2^)/3
5327 reflections	(Δ/σ)_max_ < 0.001
182 parameters	Δρ_max_ = 0.36 e Å^−^^3^
0 restraints	Δρ_min_ = −0.14 e Å^−^^3^

### Special details

**Table:**

Experimental. Bruker AXS *APEX* CCD area detector on Huber four circle diffractometer is used
Geometry. All e.s.d.'s (except the e.s.d. in the dihedral angle between two l.s. planes) are estimated using the full covariance matrix. The cell e.s.d.'s are taken into account individually in the estimation of e.s.d.'s in distances, angles and torsion angles; correlations between e.s.d.'s in cell parameters are only used when they are defined by crystal symmetry. An approximate (isotropic) treatment of cell e.s.d.'s is used for estimating e.s.d.'s involving l.s. planes.
Refinement. Refinement of *F*^2^ against ALL reflections. The weighted *R*-factor *wR* and goodness of fit *S* are based on *F*^2^, conventional *R*-factors *R* are based on *F*, with *F* set to zero for negative *F*^2^. The threshold expression of *F*^2^ > σ(*F*^2^) is used only for calculating *R*-factors(gt) *etc*. and is not relevant to the choice of reflections for refinement. *R*-factors based on *F*^2^ are statistically about twice as large as those based on *F*, and *R*- factors based on ALL data will be even larger.

### Fractional atomic coordinates and isotropic or equivalent isotropic displacement parameters (Å^2^)

**Table:**

	*x*	*y*	*z*	*U*_iso_*/*U*_eq_	
C1	0.6509 (2)	0.21826 (15)	0.99509 (11)	0.0553 (3)	
H1	0.5292	0.2328	0.9636	0.066*	
C2	0.6807 (3)	0.10113 (17)	1.07380 (11)	0.0650 (4)	
H2	0.5794	0.0373	1.0949	0.078*	
C3	0.8605 (3)	0.07815 (17)	1.12150 (11)	0.0653 (4)	
H3	0.8807	−0.0014	1.1742	0.078*	
C4	1.0085 (3)	0.17266 (18)	1.09103 (12)	0.0671 (4)	
H4	1.1283	0.1585	1.1238	0.080*	
C5	0.9801 (2)	0.28959 (16)	1.01128 (11)	0.0581 (3)	
H5	1.0830	0.3523	0.9902	0.070*	
C6	0.80051 (19)	0.31483 (12)	0.96224 (9)	0.0447 (2)	
C7	0.77651 (19)	0.44124 (13)	0.87988 (9)	0.0469 (2)	
H7	0.8947	0.4911	0.8572	0.056*	
C8	0.60545 (19)	0.49270 (12)	0.83399 (9)	0.0447 (2)	
H8	0.4831	0.4467	0.8541	0.054*	
C9	0.60632 (18)	0.62255 (12)	0.75108 (9)	0.0439 (2)	
O1	0.74616 (16)	0.70168 (10)	0.73318 (8)	0.0600 (2)	
N1	0.44089 (16)	0.65399 (10)	0.69570 (8)	0.0474 (2)	
C10	0.2872 (2)	0.56144 (15)	0.69998 (10)	0.0537 (3)	
H10	0.1398	0.6040	0.7155	0.064*	
C11	0.3327 (2)	0.41992 (15)	0.68402 (10)	0.0545 (3)	
H11	0.2124	0.3729	0.6941	0.065*	
C12	0.5480 (2)	0.32715 (12)	0.65251 (9)	0.0472 (3)	
C13	0.5818 (3)	0.17475 (15)	0.68682 (12)	0.0663 (4)	
H13	0.4678	0.1327	0.7309	0.080*	
C14	0.7817 (4)	0.08498 (16)	0.65648 (15)	0.0781 (5)	
H14	0.8016	−0.0160	0.6811	0.094*	
C15	0.9499 (3)	0.14446 (17)	0.59047 (15)	0.0727 (4)	
H15	1.0847	0.0843	0.5707	0.087*	
C16	0.9191 (3)	0.29425 (15)	0.55317 (12)	0.0622 (3)	
H16	1.0325	0.3345	0.5071	0.075*	
C17	0.7205 (2)	0.38448 (12)	0.58411 (10)	0.0512 (3)	
H17	0.7019	0.4853	0.5588	0.061*	
C18	0.4179 (3)	0.79342 (14)	0.62545 (12)	0.0628 (4)	
H181	0.4671	0.8674	0.6533	0.094*	
H182	0.2669	0.8259	0.6201	0.094*	
H183	0.5048	0.7788	0.5567	0.094*	

### Atomic displacement parameters (Å^2^)

**Table:**

	*U*^11^	*U*^22^	*U*^33^	*U*^12^	*U*^13^	*U*^23^
C1	0.0516 (7)	0.0576 (7)	0.0575 (7)	−0.0127 (5)	−0.0159 (5)	0.0013 (5)
C2	0.0691 (9)	0.0633 (8)	0.0597 (7)	−0.0183 (7)	−0.0113 (6)	0.0071 (6)
C3	0.0761 (10)	0.0640 (8)	0.0494 (6)	−0.0032 (7)	−0.0156 (6)	0.0042 (5)
C4	0.0658 (9)	0.0725 (9)	0.0631 (8)	0.0005 (7)	−0.0303 (7)	−0.0012 (6)
C5	0.0509 (7)	0.0600 (7)	0.0658 (7)	−0.0082 (5)	−0.0226 (6)	−0.0014 (6)
C6	0.0418 (5)	0.0463 (5)	0.0444 (5)	−0.0025 (4)	−0.0094 (4)	−0.0067 (4)
C7	0.0399 (5)	0.0485 (5)	0.0513 (6)	−0.0071 (4)	−0.0105 (4)	−0.0027 (4)
C8	0.0412 (5)	0.0477 (5)	0.0451 (5)	−0.0077 (4)	−0.0099 (4)	−0.0039 (4)
C9	0.0391 (5)	0.0432 (5)	0.0478 (5)	−0.0020 (4)	−0.0096 (4)	−0.0061 (4)
O1	0.0524 (5)	0.0530 (5)	0.0762 (6)	−0.0151 (4)	−0.0203 (4)	0.0048 (4)
N1	0.0443 (5)	0.0433 (4)	0.0536 (5)	−0.0013 (4)	−0.0153 (4)	−0.0032 (4)
C10	0.0359 (5)	0.0669 (7)	0.0574 (6)	−0.0076 (5)	−0.0133 (4)	−0.0011 (5)
C11	0.0476 (6)	0.0678 (7)	0.0541 (6)	−0.0262 (5)	−0.0145 (5)	0.0017 (5)
C12	0.0575 (7)	0.0446 (5)	0.0454 (5)	−0.0206 (4)	−0.0164 (5)	0.0005 (4)
C13	0.0903 (11)	0.0509 (7)	0.0635 (8)	−0.0319 (7)	−0.0235 (7)	0.0119 (6)
C14	0.1117 (14)	0.0410 (6)	0.0849 (11)	−0.0077 (7)	−0.0405 (10)	0.0048 (6)
C15	0.0796 (11)	0.0546 (7)	0.0865 (11)	0.0040 (7)	−0.0314 (9)	−0.0155 (7)
C16	0.0612 (8)	0.0542 (7)	0.0714 (8)	−0.0118 (6)	−0.0074 (6)	−0.0135 (6)
C17	0.0578 (7)	0.0398 (5)	0.0557 (6)	−0.0146 (4)	−0.0066 (5)	−0.0036 (4)
C18	0.0745 (9)	0.0452 (6)	0.0685 (8)	0.0004 (6)	−0.0289 (7)	0.0005 (5)

### Geometric parameters (Å, °)

**Table:**

C1—C2	1.3798 (19)	N1—C18	1.4566 (16)
C1—C6	1.3896 (18)	C10—C11	1.3245 (19)
C1—H1	0.9300	C10—H10	0.9300
C2—C3	1.383 (2)	C11—C12	1.471 (2)
C2—H2	0.9300	C11—H11	0.9300
C3—C4	1.366 (2)	C12—C17	1.3902 (18)
C3—H3	0.9300	C12—C13	1.3960 (17)
C4—C5	1.386 (2)	C13—C14	1.384 (3)
C4—H4	0.9300	C13—H13	0.9300
C5—C6	1.3902 (17)	C14—C15	1.367 (3)
C5—H5	0.9300	C14—H14	0.9300
C6—C7	1.4611 (16)	C15—C16	1.381 (2)
C7—C8	1.3228 (16)	C15—H15	0.9300
C7—H7	0.9300	C16—C17	1.381 (2)
C8—C9	1.4797 (16)	C16—H16	0.9300
C8—H8	0.9300	C17—H17	0.9300
C9—O1	1.2254 (15)	C18—H181	0.9600
C9—N1	1.3621 (15)	C18—H182	0.9600
N1—C10	1.4133 (17)	C18—H183	0.9600
			
C2—C1—C6	120.79 (13)	C11—C10—N1	126.33 (11)
C2—C1—H1	119.6	C11—C10—H10	116.8
C6—C1—H1	119.6	N1—C10—H10	116.8
C1—C2—C3	120.35 (14)	C10—C11—C12	128.68 (11)
C1—C2—H2	119.8	C10—C11—H11	115.7
C3—C2—H2	119.8	C12—C11—H11	115.7
C4—C3—C2	119.77 (13)	C17—C12—C13	117.46 (13)
C4—C3—H3	120.1	C17—C12—C11	122.16 (11)
C2—C3—H3	120.1	C13—C12—C11	120.30 (12)
C3—C4—C5	120.01 (13)	C14—C13—C12	121.25 (14)
C3—C4—H4	120.0	C14—C13—H13	119.4
C5—C4—H4	120.0	C12—C13—H13	119.4
C4—C5—C6	121.19 (14)	C15—C14—C13	120.15 (13)
C4—C5—H5	119.4	C15—C14—H14	119.9
C6—C5—H5	119.4	C13—C14—H14	119.9
C1—C6—C5	117.88 (11)	C14—C15—C16	119.79 (16)
C1—C6—C7	123.17 (11)	C14—C15—H15	120.1
C5—C6—C7	118.95 (11)	C16—C15—H15	120.1
C8—C7—C6	127.28 (11)	C15—C16—C17	120.21 (15)
C8—C7—H7	116.4	C15—C16—H16	119.9
C6—C7—H7	116.4	C17—C16—H16	119.9
C7—C8—C9	120.86 (11)	C16—C17—C12	121.10 (12)
C7—C8—H8	119.6	C16—C17—H17	119.5
C9—C8—H8	119.6	C12—C17—H17	119.5
O1—C9—N1	120.39 (11)	N1—C18—H181	109.5
O1—C9—C8	122.32 (10)	N1—C18—H182	109.5
N1—C9—C8	117.28 (10)	H181—C18—H182	109.5
C9—N1—C10	125.32 (10)	N1—C18—H183	109.5
C9—N1—C18	118.41 (11)	H181—C18—H183	109.5
C10—N1—C18	116.27 (10)	H182—C18—H183	109.5
			
C6—C1—C2—C3	0.1 (2)	O1—C9—N1—C18	−7.99 (17)
C1—C2—C3—C4	0.5 (2)	C8—C9—N1—C18	170.62 (10)
C2—C3—C4—C5	−1.1 (2)	C9—N1—C10—C11	−53.69 (19)
C3—C4—C5—C6	1.1 (2)	C18—N1—C10—C11	126.00 (14)
C2—C1—C6—C5	−0.1 (2)	N1—C10—C11—C12	−3.9 (2)
C2—C1—C6—C7	−179.54 (12)	C10—C11—C12—C17	−34.6 (2)
C4—C5—C6—C1	−0.5 (2)	C10—C11—C12—C13	148.71 (14)
C4—C5—C6—C7	178.95 (12)	C17—C12—C13—C14	1.9 (2)
C1—C6—C7—C8	8.2 (2)	C11—C12—C13—C14	178.75 (13)
C5—C6—C7—C8	−171.22 (12)	C12—C13—C14—C15	−1.0 (2)
C6—C7—C8—C9	−179.88 (10)	C13—C14—C15—C16	−0.7 (3)
C7—C8—C9—O1	−11.86 (18)	C14—C15—C16—C17	1.3 (2)
C7—C8—C9—N1	169.57 (11)	C15—C16—C17—C12	−0.3 (2)
O1—C9—N1—C10	171.70 (11)	C13—C12—C17—C16	−1.29 (19)
C8—C9—N1—C10	−9.70 (17)	C11—C12—C17—C16	−178.04 (12)
